# Application of Bioavailability Models to Derive Chronic Guideline Values for Nickel in Freshwaters of Australia and New Zealand

**DOI:** 10.1002/etc.4885

**Published:** 2020-11-17

**Authors:** Jenny Stauber, Lisa Golding, Adam Peters, Graham Merrington, Merrin Adams, Monique Binet, Graeme Batley, Francesca Gissi, Kitty McKnight, Emily Garman, Ellie Middleton, Jennifer Gadd, Chris Schlekat

**Affiliations:** ^1^ Commonwealth Scientific and Industrial Research Organisation Land and Water, Lucas Heights, New South Wales Australia; ^2^ WCA Environment, Faringdon, Oxfordshire United Kingdom; ^3^ Commonwealth Scientific and Industrial Research Organisation Oceans and Atmosphere, Lucas Heights, New South Wales Australia; ^4^ NiPERA Durham North Carolina USA; ^5^ National Institute of Water and Atmospheric Research Auckland New Zealand

**Keywords:** Biotic ligand model, Multiple linear regressions, Water quality criteria, Metals

## Abstract

There has been an increased emphasis on incorporating bioavailability‐based approaches into freshwater guideline value derivations for metals in the Australian and New Zealand water quality guidelines. Four bioavailability models were compared: the existing European biotic ligand model (European Union BLM) and a softwater BLM, together with 2 newly developed multiple linear regressions (MLRs)—a trophic level‐specific MLR and a pooled MLR. Each of the 4 models was used to normalize a nickel ecotoxicity dataset (combined tropical and temperate data) to an index condition of pH 7.5, 6 mg Ca/L, 4 mg Mg/L, (i.e., approximately 30 mg CaCO_3_/L hardness), and 0.5 mg DOC/L. The trophic level‐specific MLR outperformed the other 3 models, with 79% of the predicted 10% effect concentration (EC10) values within a factor of 2 of the observed EC10 values. All 4 models gave similar normalized species sensitivity distributions and similar estimates of protective concentrations (PCs). Based on the index condition water chemistry proposed as the basis of the national guideline value, a protective concentration for 95% of species (PC95) of 3 µg Ni/L was derived. This guideline value can be adjusted up and down to account for site‐specific water chemistries. Predictions of PC95 values for 20 different typical water chemistries for Australia and New Zealand varied by >40‐fold, which confirmed that correction for nickel bioavailability is critical for the derivation of site‐specific guideline values. *Environ Toxicol Chem* 2021;40:100–112. © 2020 The Authors. *Environmental Toxicology and Chemistry* published by Wiley Periodicals LLC on behalf of SETAC.

## Graphical abstract

Derivation of bioavailability‐based guidelines for nickel in freshwaters after normalization of ecotoxicity data using trophic level‐specific multiple linear regression (MLRs). SSD** =** species sensitivity distribution.

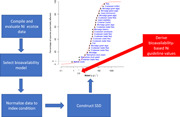

## INTRODUCTION

It is well known that water chemistry parameters such as hardness, pH, and dissolved organic carbon (DOC), influence metal bioavailability and consequently toxicity to freshwater biota, either through complexation in solution (e.g., DOC) or through competition with the biotic ligand (e.g., hardness, H^+^ ions). For the past 2 decades, hardness algorithms have routinely been used in Australia and New Zealand for the metals cadmium, chromium, copper, lead, nickel, and zinc, with all toxicity data normalized to 30 mg CaCO_3_/L (Australian and New Zealand Environment and Conservation Council/Agriculture and Resource Management Council of Australia and New Zealand 2000). These hardness algorithms were also used to correct guideline values for site‐specific hardness conditions. The algorithms use relationships from North American acute fish and invertebrate toxicity data adopted from the US Environmental Protection Agency (USEPA 1995). Since then, considerable research has shown the limitations of these algorithms. For example, Markich et al. (2005) showed that hardness has either no or limited effect on chronic copper toxicity to a variety of sensitive species including a bacterium, a microalga, and a cladoceran (no fish). This is important given the emphasis placed within Australia and New Zealand on chronic ecotoxicity data for guideline derivation (Warne et al. 2018). Other criticisms of the hardness algorithms are that they exclude softer waters typical of some parts of Australia, and that they are derived from a limited range of species and are then extrapolated to all species.

Other water chemistry parameters such as pH and DOC play an equally, if not more important role in controlling metal bioavailability, and these toxicity‐modifying factors have been incorporated into bioavailability tools such as the biotic ligand model (BLM; Di Toro et al. 2001). The BLMs are quasi‐mechanistic models that use a range of parameters (Ca, Mg, Na, K, SO_4_, Cl, pH, DOC, temperature, and alkalinity) to predict metal accumulation at an undefined site of toxic action in the organism and the corresponding toxic effects (Di Toro et al. 2001; Mebane et al. 2020a). Certain BLMs (acute and some chronic) have now been developed for Cu, Zn, Ni, Pb, Ag, Cd, and Al in freshwaters (Mebane et al. 2020a). Bioavailability‐based water quality criteria for copper in the United States (US Environmental Protection Agency 2007) and environmental quality standards for nickel in the European Union (European Commission 2011) were derived using BLMs. A simplified bioavailability tool called Bio‐met (2019) that requires a reduced number of water chemistry parameters was developed to determine compliance with the European Union nickel environmental quality standard. Bio‐met can be used either to adjust guideline values based on specific water chemistries, or to estimate bioavailable concentrations of metals in environmental samples (Peters et al. 2016). These models have been used to show that in general, the toxicity of nickel to freshwater species increases with increasing pH, decreasing hardness, and decreasing DOC (Peters et al. 2018).

At present, no bioavailability models have been endorsed to derive bioavailability‐based guideline values for Australia and New Zealand, although the guideline value derivation methodology guidance (Warne et al. 2018) has been recently revised to allow for consideration of a range of bioavailability‐based tools including the BLM. Additional emphasis has been placed on the appropriate validation of such tools using water quality conditions and species relevant for Australia and New Zealand.

Considerable work has been conducted to determine the validity of the nickel BLMs developed for waters in Europe (Nys et al. 2016), waters in North America (Schlekat et al. 2010), and waters in Australia and New Zealand (Peters et al. 2018). Peters et al. (2018) modified chronic nickel BLMs previously developed for the freshwater alga *Pseudokirchneriella subcapitata*, 2 invertebrates *Daphnia magna* and *Ceriodaphnia dubia*, and one fish *Oncorhynchus mykiss* (Nys et al. 2016) for application to the softer waters (<50 mg CaCO_3_/L) commonly found in Australia. Due to the increased competitive effects of calcium and magnesium with nickel for binding to the biotic ligand in very soft waters, modifications were made to the existing BLM by increasing the stability constants (log *K* values) for calcium and magnesium from the 3.5 used in the original models to 5 in the softwater BLM.

Another approach incorporating bioavailability for metals that has gained considerable attention from regulatory agencies in recent years uses multiple linear regressions (MLRs) in a similar manner to BLMs; for example, in Environment Canada's revised draft water quality guideline value for zinc (Canadian Council of Ministers of the Environment 2018) and in the USEPA's freshwater aluminum ambient water quality criteria (US Environmental Protection Agency 2018). Similarly, MLRs are used in the Australian ecological investigation levels for nickel in contaminated soils (National Environment Protection Council 2013). Similar to the BLM approach, toxicity data from single species can be used to develop relationships with water quality parameters, or data for multiple species can be pooled to select the best MLR, provided that water chemistry effects on toxicity are similar across species. It has been suggested that MLR approaches may assist the transition from algorithms focusing on single toxicity‐modifying factors (e.g., hardness) to more complex BLM approaches considering multiple factors for metal guideline value derivations (Brix et al. 2017, 2020; Garman et al. 2020).

In Australia and New Zealand, the freshwater nickel guideline value for 95% species protection is currently 11 µg Ni/L. However, this is based on chronic toxicity data for dissolved nickel in synthetic waters low in DOC, normalized to a hardness of 30 mg CaCO_3_/L, and within the pH range 6.3 to 7.7 (Australian and New Zealand Environment and Conservation Council/Agriculture and Resource Management Council of Australia and New Zealand 2000). Test pH has been shown to also influence nickel bioavailability, with toxicity generally increasing with increasing pH (Deleebeeck et al. 2009). This guideline value was based on just 7 species from 4 taxonomic groups (as defined in Warne et al. 2018) using a species sensitivity distribution (SSD) method. There is now a large database of high‐quality nickel effects data for both tropical and temperate freshwater biota (Scientific Committee on Health and Environmental Risks 2011; Binet et al. 2018). This provides an opportunity to revise the Australian and New Zealand guideline values for nickel and to incorporate bioavailability‐based approaches using either existing BLMs or through the development of entirely new MLRs.

Until recently, no MLRs had been published and validated for nickel in freshwaters. Therefore, we developed trophic level‐specific MLRs for a freshwater alga *P. subcapitata*, an aquatic plant (*Lemna minor*), 3 invertebrates (*D. magna, C. dubia* and an Australian clone of *Hydra viridissma*), and one fish *Pimephales promelas*, as well as a pooled MLR (Peters et al. 2021). In contrast to North American MLRs for other metals, these MLR models were based on 10% effect concentration (EC10) values rather than median effect concentration (EC50) values, because EC10 values are used in Australia and New Zealand in SSDs to derive guideline values. In the present study we compared these new MLRs with 2 existing nickel BLMs: a European BLM (European Union BLM; Nys et al. 2016) and a softwater BLM (Peters et al. 2018), using a modified scoring method of Van Genderen et al. (2020) to assess model accuracy, taxa representation, water chemistry coverage, and ease of use. The best models were used to normalize high‐quality chronic ecotoxicity data to an index condition to derive new guideline values for nickel. The models were also tested using a range of natural waters with varying water quality parameters to assess their performance in deriving site‐specific guideline values in Australia and New Zealand.

## MATERIALS AND METHODS

### Nickel ecotoxicity data compilation

Databases of chronic nickel toxicity, including all water quality parameters, for tropical and temperate exotic and native freshwater species were compiled and quality assessed following the method of Warne et al. (2018). “Tropical” was defined as test species isolated from tropical regions and/or whose natural geographic distribution was between the Tropic of Cancer and the Tropic of Capricorn, and the test was carried out at ≥25 °C. “Temperate” species included all species outside this region and where tests were carried out at <25 °C. All data were quality checked using a data quality checklist (Australian and New Zealand Governments 2018; Warne et al. 2018). Only chronic data scoring ≥50% and with measured dissolved nickel concentrations were used as the final nickel freshwater ecotoxicity dataset. To achieve a score of ≥50%, criteria to be met in the checklist of Warne et al. (2018) included, but were not limited to, use of appropriate controls, replication of controls and contaminant concentrations, inclusion of reference toxicant, stated test acceptability criteria, description of test organism (e.g., life‐stage, length, mass, age, etc,), measurement of contaminant concentrations, measurement of water quality parameters, and use of the appropriate statistical method to determine toxicity.

The temperate dataset included 20 species from 6 taxonomic groups (as defined by Warne et al. 2018), and the tropical dataset comprised 24 species from 6 taxonomic groups (Supplemental Data, Table S1). The combined temperate and tropical dataset included 44 species from 9 taxonomic groups. Not all these species had sufficient water chemistry data available, so only 26 species of the combined temperate and tropical dataset were normalized using the BLMs and MLRs and so included in the SSDs.

### Bioavailability normalizations and SSDs

The following four different methods were used to generate SSDs from the ecotoxicity datasets.

#### No bioavailability correction—full dataset

Chronic effects data for temperate, tropical, and combined temperate and tropical species were not normalized to a standard set of water chemistry parameters.

#### No bioavailability correction—censored dataset

Boundaries on water chemistry parameters were established so that only ecotoxicity test data conducted under relatively high nickel bioavailability conditions with DOC ≤1 mg/L, hardness ≤50 mg CaCO_3_/L, and pH in the range of 6 to 9 were included in the SSDs. The SSDs for temperate, tropical, and temperate/tropical combined datasets were compared.

#### MLRs

The MLRs for 4 different trophic levels (algae, aquatic plants, invertebrates, and fish), as well as a pooled MLR model that combined the trophic‐level MLRs (Peters et al. 2021) were used to normalize the ecotoxicity data for the combined tropical/temperate dataset only (26 species). These MLRs are shown in Table 1.

**Table 1:** Trophic level‐specific and pooled multiple linear regression (MLR)^a^

Trophic level	MLR
Algae	Log_e_(EC10) = sensitivity + 0.28.log_e_(DOC) + 0.50.log_e_(Mg) − 0.20.pH
Aquatic plants	Log_e_(EC10) = sensitivity + 0.96.log_e_(DOC) − 1.44.pH
Invertebrates	Log_e_(EC10) = sensitivity + 2.09.log_e_(DOC) + 0.19.log_e_(Ca) + 0.40.log_e_(Mg) − 0.40.pH − 0.24.log_e_(DOC).pH
Fish	Log_e_(EC10) = sensitivity − 1.05.log_e_(DOC) + 3.55.log_e_(Mg) − 0.07.pH + 0.19.log_e_(DOC).pH − 0.42.log_e_(Mg).pH
Pooled	Log_e_(EC10) = sensitivity + 2.81.log_e_(DOC) − 0.16.log_e_(Ca) + 0.81.log_e_(Mg) − 0.57.pH − 0.43.log_e_(DOC).pH + 0.27.log_e_(DOC).log_e_(Mg)

^a^ From Peters et al. 2021. Note that the parameters in the MLR equations have been rounded to 2 decimal places.

EC10 = 10% effect concentration; DOC = dissolved organic carbon.

#### Combined BLMs

Existing chronic nickel BLMs (European Union BLM [Nys et al. 2016] and softwater BLM [Peters et al. 2018]) for 3 taxonomic groups (algae, invertebrates, and fish) were applied to normalize the chronic ecotoxicity data for the combined tropical/temperate dataset only (26 species). First the European Union BLM was used to normalize all the ecotoxicity data generated and is termed “European Union BLM” in the text and Table 2. Second, the softwater BLM was used to normalize all the ecotoxicity data, including data that were outside its hardness range, and is termed “softwater BLM.” Third, the European Union/softwater BLMs were combined as follows: The European Union BLM was used only to normalize the ecotoxicity data tested at a hardness of >50 mg CaCO_3_/L, and these data were then combined with the softwater BLM‐normalized data for tests <50 mg CaCO_3_/L hardness. This dataset is termed “combined BLMs.” The disadvantage of combining the 2 BLMs is that 2 ecotoxicity databases are used—one with data on <50 mg/L hardness and one with data on >50 mg/L hardness. This comparison was therefore only done to score the models and was not used for any further estimates after the scoring process.

**Table 2:** Water chemistry boundaries for each model^a^

Model	pH	DOC (mg/L)	Ca (mg/L)	Mg (mg/L)	Calculated Hardness (mg CaCO_3_/L)
Algal MLR	6.0–8.0	0.3–26	3–144	3–115	20–481
Plant MLR	6.9–8.3	0.7–7.1	3–72	0.8–21	12–250
Invertebrate MLR	5.9–8.2	0.3–17	0.1–88	0.2–72	1–316
Fish MLR	5.5–8.5	0.5–18	3.7–110	1.1–286	14–1450
Trophic level‐specific MLR	5.5–8.5	0.3–26	0.1–144	0.2–286	1–1450
Pooled MLR	6.0–8.0^b^	0.3–26	0.1–110	0.2–286	1–1450
European Union BLM	6.5–8.7	<0.5–26^c^	2–88	—d	8–250
Softwater BLM	5.5–8.7	<0.5–26^c^	0.1–10	—	1–50

^a^ From Peters et al. 2021.

^b^ Note that this was the approximate pH range for the algal and plant data, with a larger pH range for invertebrates and fish.

^c^ BLMs do not have boundary conditions for DOC, rather, this is the range of DOC in the tests used to derive and validate the BLMs.

^d^ Boundaries for Mg in BLMs are implied, although not specified, by the boundaries for Ca, and because both Ca and Mg have the same effect on nickel toxicity (including identical log *K* values for binding to the biotic ligand).

DOC = dissolved organic carbon; MLR = multiple linear regression; BLM = biotic ligand model.

The water chemistry boundaries for each of the models, based on the model development datasets, are shown in Table 2. Water chemistry boundaries for the development datasets are given in the Supplemental Data, Table S2.

All SSDs were derived using the Burrlioz Ver 2.0 software as recommended by Australian and New Zealand Governments (2018). Concentrations to protect 80% (PC80), 90% (PC90), 95% (PC95), and 99% (PC99) of species were determined, together with confidence limits. The PC95 is the guideline value usually applied to slightly‐to‐moderately disturbed systems in Australia and New Zealand, whereas the PC99 is applied to systems of high ecological value.

### Scoring of models

Traditionally, bioavailability models have been evaluated based on observed versus predicted ecotoxicity, with a rule of thumb being that models showing a <2 difference are considered to be effective. Van Genderen et al. (2020) introduced a more rigorous comparative approach. Each model was therefore evaluated using a modified method of Van Genderen et al. (2020) to select the most suitable model with which to normalize the ecotoxicity data to generate SSDs and guideline values for nickel. Trophic level‐specific MLRs, the pooled MLRs, the European Union BLM, and the softwater BLM were scored according to how well the model represented the water chemistry and taxa in the ecotoxicity dataset, and how well the model predicted toxicity based on residual factors.

The scoring derived from van Genderen et al. (2020) was undertaken as follows.

#### Water chemistry coverage

Relative to the range of water chemistries used in the model development, a score of 1 was assigned to toxicity tests with water quality reported within the model boundaries and 0 for water quality outside the boundary range. The DOC and pH, together with Ca and Mg concentrations, were examined. Sometimes only one parameter was out of range, so best professional judgement was used to determine the final score. Generally, if only one parameter was slightly out of range, it was scored as a 1.

#### Taxa coverage

Each species in the ecotoxicity dataset was assigned a scaled score based on whether it was taxonomically similar to the species used to derive the model, that is, a within‐biological organization comparison (Van Genderen et al. 2020): 0 (outside Kingdom), 1 (Kingdom), 2 (Phylum), 3 (Class), 4 (Order), 5 (Family), 6 (Genus), and 7 (Species).

#### Nonproportional approach

Water quality and taxa coverage scores were summed for each test and ranked as good (score = 6–8), fair (score = 3–5), or poor (score = 0–2), according to the original approach of Van Genderen et al. (2020). The scores were summed for each trophic level and summed overall. This approach could lead, however, to overweighting of the species for which there were a larger number of tests, so a proportional approach was also used for comparison.

#### Proportional approach

The number of good scores was divided by the total number of tests for that trophic level. This results in lower scores (not scaled) than the nonproportional approach.

#### Model performance

Residual factors were calculated as the maximum divided by the minimum value of predicted versus observed EC10 values (always a positive number), and then plotted as cumulative distribution functions for each model. When observed versus predicted EC10 values were within a factor of 2, this was equivalent to a residual factor of ≤2. Higher residual factor values indicate poorer agreement between predicted and observed toxicity (Meyer et al. 2018). The percentage of data with residual factor values ≤2 was determined for each model.

#### Obtaining relative scores

The total number of good scores (nonproportional approach) or number of good scores as a proportion of the total number of tests for that trophic level (proportional approach), were multiplied by the residual factor to obtain relative scores.

#### Ranking of relative scores

Relative scores were ranked (1 being best) by trophic level and overall to assist in the selection of the best model to normalize the ecotoxicity data.

### Using the models to normalize the nickel ecotoxicity dataset to an index condition

As part of the model comparison to guide selection of the most appropriate model for nickel guideline value derivation, each model was used to normalize the nickel ecotoxicity dataset to an index condition. The index condition is a specific combination of water chemistry parameters, usually representing relatively high metal bioavailability conditions. The index condition for Australia and New Zealand was agreed to by a panel of project advisors to be pH 7.5, 6 mg Ca/L, 4 mg Mg/L, (i.e., hardness of ~30 mg CaCO_3_/L), and 0.5 mg/L DOC.

Of the 44 species in the dataset, 26 species had sufficient water chemistry data to be included in the final normalized SSDs. The SSDs for nickel ecotoxicity data were normalized with each of the 4 models, that is, each of the models was used to predict the EC10 values for each species at the index chemistry condition.

### Applying the models in different water chemistry conditions

Typical ranges of water chemistry parameters for Australia and New Zealand are given by Batley et al. (2018) and are summarized in Table 3.

**Table 3:** Ranges for general water quality parameters in Australia and New Zealand^a^

		Range (10th and 90th percentiles)	
Parameter	Unit	Australia	New Zealand
pH	—	6.0–7.6	7.2–8.2
Ca	mg Ca/L	0.4–45	4.2–17.4
Mg	mg Mg/L	0.5–30	0.7–3.6
Hardness	mg CaCO_3_/L	3–236	16–56
DOC	mg/L	2–12	2.6–15

^a^ From Batley et al. 2018.

DOC = dissolved organic carbon.

To assess how nickel guideline values would vary with different water chemistries and with the choice of bioavailability models, databases were examined to select 20 waters spanning a wide range of water chemistry values and geographic locations and potential bioavailability conditions for nickel exposures (Table 4). These included 10 natural waters from Australia (tropical and temperate) and 10 natural waters from New Zealand. Mean water chemistry parameters were used to calculate bioavailability‐normalized protective concentrations for each water. One natural water (Ovens River) was also adjusted to pH 8.2 and 0.5 mg/L DOC, so 2 sets of protective concentration values were available for this river. The nickel ecotoxicity data were then normalized to these 20 different water chemistries using the 4 models (trophic level‐specific MLR, pooled MLR, European Union BLM, and softwater BLM), and the protective concentration values from each SSD were compared. The softwater BLM was only applied to 7 natural Australian waters and 9 natural New Zealand waters with <50 mg CaCO_3_/L hardness.

**Table 4:** Water quality parameters for each of the 20 natural waters from Australia and New Zealand

Water	pH	DOC (mg/L)	Ca (mg/L)	Mg (mg/L)	Calculated hardness (mg CaCO_3_/L)
Australian waters					
Appletree Creek	4.6	12	0.25	1.5	7
Magela Creek	6.1	3	0.25	0.25	2
Ovens River	6.9	3	3.1	2.9	20
Ovens River adj.	8.2	0.5	3.1	2.9	20
Minamurra River	7.1	7	6.6	5.6	40
Tea Tree	7.3	10	2.1	4.7	25
Lake Eacham	7.4	1.4	1.8	2.3	14
Woronora River	7.6	4	2.5	3.5	21
Wellington	7.9	5	11	7.4	58
Sandy Creek	7.2	21	21	16	117
Milang	8.8	23	23	17	128
New Zealand waters					
Cascade Stream	7.8	0.5	6.9	4.5	36
Waipaoa River	8.2	2.9	68	6.8	198
Rangataiki River	7.0	1.5	5.1	1.9	21
Ohinemuri River	8.3	1.9	3.9	1.9	18
Hurunui River	8.2	1.9	9.9	1.4	31
Haast River	7.7	0.8	14	0.75	37
Mangapouri Stream	7.0	5.8	9.2	5.2	44
Porirua Stream	7.5	2.2	8.9	5.1	43
Mokotua Stream	4.4	33	1.3	2.9	15
Carran Creek	6.5	18	11	5.0	48
Index condition	7.5	<0.5	6	4	30

DOC = dissolved organic carbon.

## RESULTS

The nickel chronic ecotoxicity database used for all normalizations with the models is provided in the Supplemental Data, Table S1.

### SSDs with no normalization

For the unbounded full dataset (i.e., including all tests with or without associated water chemistry reported), separate SSDs were compiled for temperate data (20 species from 6 taxonomic groups), tropical data (24 species from 6 taxonomic groups), and combined temperate and tropical data (44 species from 9 taxonomic groups). These SSDs are presented in Supplemental Data, Figure S1, with protective concentration values and 95% confidence limits for 80, 90, 95, and 99% species protection given in Table 5.

**Table 5:** Protective concentrations (PCs; µg Ni/L) from bounded (dissolved organic carbon ≤1 mg/L, hardness ≤50 mg CaCO_3_/L, and pH in the range 6–9) and unbounded (all data) nickel freshwater datasets with no correction for bioavailability^a^

Dataset		PC99	PC95	PC90	PC80
Temperate	Unbounded	0.94 (0.09–5.5)	2.8 (0.92–8.8)	4.8 (2.3–12)	9.0 (4.3–21)
Tropical	Unbounded	0.73 (0.08–7.3)	2.7 (0.84–11)	5.1 (2–15)	10 (4.7–21)
Combined	Unbounded	0.77 (0.17–3.9)	2.7 (1.2–6.8)	4.9 (2.5–10)	9.7 (5.5–17)
Temperate	Bounded	0.44 (0.04–3.4)	1.6 (0.51–5.7)	2.8 (1.3–8.3)	5.4 (2.3–13)
Tropical	Bounded	2.1 (0.14–8.0)	4.9 (1.6–11)	7.3 (4–15)	12 (6.8–23)
Combined	Bounded	0.73 (0.12–3.9)	2.5 (1.1–6.4)	4.4 (2.4–8.7)	8.1 (4.8–14)

^a^ Confidence limits in parentheses.

The PC95 values were similar for tropical (2.7 µg Ni/L), temperate (2.8 µg Ni/L), and combined tropical and temperate data (2.7 µg Ni/L), with good curve fits (Supplemental Data, Figure S1). This is in agreement with the findings of Peters et al. (2019), who showed that there was no significant difference between tropical and temperate freshwater SSDs for nickel, despite the different representation of taxonomic groups.

**Figure 1:**
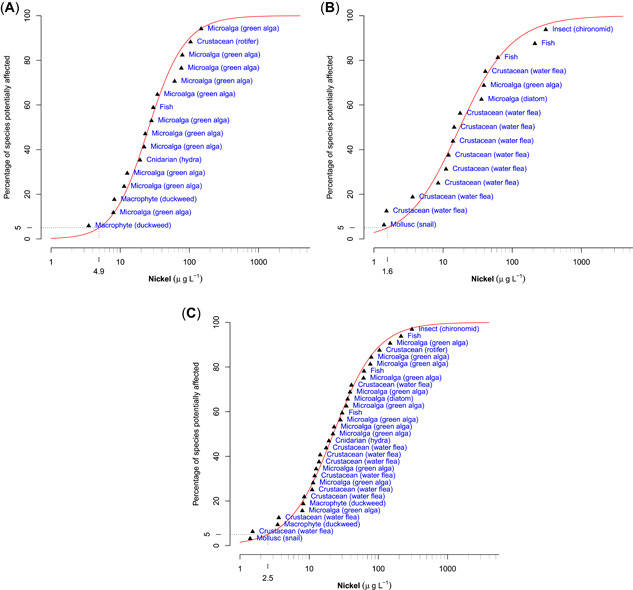
Species sensitivity distributions (SSDs) for bounded datasets: (**A**) tropical, (**B**) temperate, and (**C**) combined tropical and temperate.

A subset of each of the temperate and tropical unbounded datasets (censored dataset) was also compiled. Only data with DOC ≤1 mg/L, hardness ≤50 mg CaCO_3_/L, and pH in the range of 6 to 9 were included, to reduce variations in sensitivity resulting from differences in water chemistry. Separate SSDs were constructed for temperate data (15 species from 5 taxonomic groups), tropical data (16 species from 5 taxonomic groups), and combined tropical and temperate data (31 species from 7 taxonomic groups). These SSDs are presented in Figure 1, with protective concentration values and 95% confidence limits for 80, 90, 95, and 99% given in Table 5. Comparisons are confounded to some extent by the limited and different taxonomic groups in the tropical dataset (mostly microalgae and macrophytes) compared with the temperate dataset (mostly invertebrates). The PC95 values were 4.9, 1.6, and 2.5 µg Ni/L for the tropical, temperate, and combined temperate and tropical datasets, respectively, and their associated confidence limits overlapped.

The PC95 values for the bounded and unbounded combined datasets were also similar, with values of 2.5 and 2.7 µg Ni/L, respectively. Given the wide confidence limits, these values could be rounded up to one significant figure, that is, 3 µg Ni/L, if they were to be applied as guideline values for slightly‐to‐moderately disturbed systems.

### Model scores

**Figure 2:**
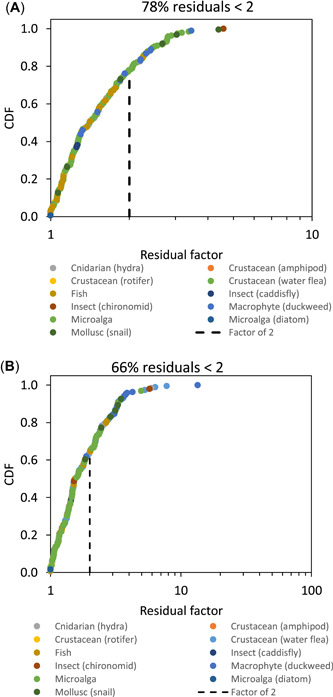
Cumulative distribution function (CDF) plots of residuals for (**A**) trophic level‐specific multiple linear regressions (MLRs) and (**B**) pooled MLR.

**Figure 3:**
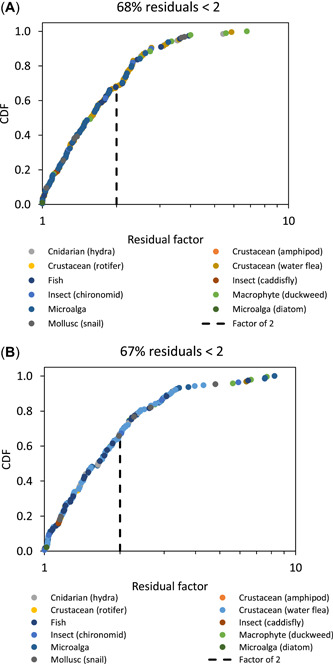
Cumulative distribution function (CDF) plots of residuals for (**A**) European Union biotic ligand model (BLM) and (**B**) soft water BLM (all data).

Figures 2 and 3 show the plots of the residual factors for each of the 4 models applied to all data: 1) trophic level‐specific MLRs, that is, algal MLR applied to algae in the ecotoxicity database, aquatic plant MLR applied to aquatic plants, invertebrate MLR applied to invertebrates, and fish MLR applied to fish (Figure 2A); 2) pooled MLR applied to all taxa (Figure 2B); 3) European Union BLM, all data (Figure 3A); and 4) softwater BLM, all data (Figure 3B).

The trophic level‐specific MLR resulted in 78% of the normalized data being within a factor of 2 of the observed data, which was substantially better than the pooled MLR, which had 66% of the normalized data within a factor of 2 (Figure 2A and B). Both the European Union BLM and the softwater BLM gave results similar to each other, with 68 and 67% of residual factors within a factor of 2, respectively (Figure 3A and B).

Using the combined BLMs (i.e., the European Union BLM for data at >50 mg/L CaCO_3_ hardness together with the softwater BLM for data at hardness <50 mg CaCO_3_/L) improved the residual factors, with 75% of the predicted data within a factor of 2 of the observed data (not shown). If the softwater BLM was only used to adjust data with a hardness <50 mg CaCO_3_/L, 76% of the residual factors were within a factor of 2 (not shown). However, this limited the available dataset to just 82 tests with 19 species, compared with 190 tests with 26 species in the full dataset.

The final scores and rankings for each of the models using both nonproportional and proportional approaches are given in the Supplemental Data, Tables S3 and S4.

Using the nonproportional approach (original method) and the whole dataset, the trophic level‐specific MLR scored the best overall (119 relative score), followed by the other models: pooled MLR (94 relative score), European Union BLM (88 relative score), and softwater BLM (86 relative score, 35 relative score when using data in softwater only), which were similar to each other. If the combined BLMs were used, the relative score improved from 88 to 97. The high trophic level‐specific MLR score was not surprising, given that the trophic level‐specific MLR was developed using an additional local invertebrate species (*Hydra viridissima*) compared with the BLMs, and was trophic level‐specific compared with the pooled MLR.

Using the proportional approach, the values were much lower because each score was divided by the number of tests. For the whole nickel ecotoxicity dataset, the trophic level‐specific MLR again scored the highest (0.63 relative score), followed by the pooled MLR (0.50 relative score), European Union BLM (0.46 relative score), and softwater BLM (0.45 relative score, 0.43 relative score for softwater only data). If the combined BLMs were used, the relative score improved from 0.46 to 0.51.

### Deriving guideline values at the index condition

Of the 44 species in the combined tropical and temperate nickel ecotoxicity dataset, 26 species had sufficient water chemistry data to be used in a set of index condition SSDs. A summary of the ecotoxicity data for the combined tropical and temperate SSD at the index condition is shown in Table 6, with the SSD in the Supplemental Data, Figure S2.

**Table 6:** Summary of chronic toxicity lethal and effect concentration (LC/EC10) values normalized to the index condition (pH 7.5, 6 mg Ca/L, 4 mg Mg/L (i.e., hardness of ~30 mg CaCO_3_/L) and 0.5 mg/L dissolved organic carbon) used to derive the guideline values for nickel

Taxonomic group	Species	Life stage	Duration	Toxicity endpoint	Normalized EC10 (µg Ni/L)
Diatom	*Navicula pelliculosa*	—	72 h	Growth	32.7
Green microalga	*Chlorella* sp. (Kakadu isolate)^a^	—	72 h	Growth rate	142
Green microalga	*Chlorella* sp. 12 (PNG isolate)^a^	—	72 h	Growth rate	18.2
Green microalga	*Monoraphidium arcuatum* a	—	72 h	Growth rate	81
Green microalga	*Nannochloropsis* sp.^a^		72 h	Growth rate	12.5
Green microalga	*Pediastrum duplex* a	—	72 h	Growth rate	108
Green microalga	*Pseudokirchneriella subcapitata*	—	72 h	Growth rate	54.6
Macrophyte (duckweed)	*Lemna aequinoctialis* a	—	96 h	Growth (yield)	2.78
Macrophyte (duckweed)	*Lemna minor* a	—	7 d	Growth rate	7.94
Crustacean (rotifer)	*Brachionus calyciflorus* a	Neonates	48 h	Growth rate	217
Crustacean (amphipod)	*Hyalella azteca*	7–8 d old	14 d	Survival	32.5
Crustacean (cladoceran)	*Alona affinis vannamei*	Neonates	16 d	Survival	30.7
Crustacean (cladoceran)	*Ceriodaphnia dubia*	Neonates	7 d	Reproduction	2.78
Crustacean (cladoceran)	*Ceriodaphnia pulchella*	Neonates	17 d	Reproduction	23.3
Crustacean (cladoceran)	*Ceriodaphnia quadrangula*	Neonates	17 d	Reproduction	17.2
Crustacean (cladoceran)	*Daphnia longispina*	Neonates	21 d	Reproduction	66.9
Crustacean (cladoceran)	*Daphnia magna*	Neonates	21 d	Reproduction	17.9
Crustacean (cladoceran)	*Peracantha truncata*	Neonates	17 d	Reproduction	30.4
Crustacean (cladoceran)	*Simocephalus serrulatus*	Neonates	17 d	Reproduction	48.1
Crustacean (cladoceran)	*Simocephalus vetulus*	Neonates	21 d	Reproduction	24.6
Gastropod	*Lymnaea stagnalis*	<24 h old	30 d	Growth	0.75
Cnidarian	*Hydra viridissima* a	—	96 h	Growth (yield)	57.1
Insect (chironomid)	*Chironomus tentans*	Larvae	10 d	Growth	104
Insect (caddisfly)	*Clistoronia magnifica*	Larvae	19 d	Survival	60.3
Fish	*Melanotaenia splendida splendida* a	Embryos	12 d	Hatching	22.9
Fish	*Pimephales promelas*	Swim‐up fry	17 d	Survival	273

^a^ Tropical species.

PNG = Papua New Guinea.

Each of the 4 models was used to convert the ecotoxicity data for each of these 26 species to equivalent EC10 values at the index water chemistry condition of pH 7.5, 6 mg Ca/L, 4 mg Mg/L (i.e., hardness of ~30 mg CaCO_3_/L), and 0.5 mg/L DOC. These normalized data were then plotted in new SSDs. The PC99, PC95, PC90, and PC80 values obtained for the SSD for each model are shown in Table 7. Note that the trophic level‐specific MLR applied the algal MLR to chronic algal data, the aquatic plant MLR to chronic duckweed data, the invertebrate MLR to chronic invertebrate data, and the fish MLR to chronic fish data, and then combined these normalized EC10 values into one SSD to estimate the protective concentration values.

**Table 7:** Protective concentrations (PCs; µg/L) for nickel at the index condition (pH 7.5, 6 mg Ca/L, 4 mg Mg/L, and 0.5 mg/L dissolved organic carbon) from species sensitivity distributions using data normalized using each of the 4 models

Model	PC99	PC95	PC90	PC80
Trophic level‐specific MLR	0.44 (0.03–6.4)	2.6 (0.52–10)	5.4 (1.7–15)	12 (5.3–22)
Pooled MLR	1.8 (0.20–9.9)	5.2 (1.6–14)	8.4 (3.6–17)	14 (8.3–24)
European Union BLM	0.82 (0.06–5.3)	2.5 (0.69–7.3)	4.1 (1.7–9.0)	7.3 (4.0–12)
Soft water BLM	0.57 (0.04–5.6)	2.2 (0.55–7.9)	4.1 (1.5–9.8)	7.9 (4.0–14)

BLM = biotic ligand model; MLR = multiple linear regression.

All 4 models gave similar normalized SSDs and similar estimates of the protective concentrations (within a factor of 2 except for PC99 values), with overlapping confidence limits. The pooled MLR, however, consistently gave the highest protective concentration values (i.e., was the least protective at this index condition). The factor of 2 is an acceptable level of variation because any less would be within the normal range of variability of ecotoxicity tests conducted at different laboratories or at different times on the same species (Peters et al. 2018). Other validation studies have also generally aimed for predictions to be within a factor of 2 of the observed result, although this approach has most commonly been applied to median effect levels from acute studies (Meyer et al. 2018). Given that EC10 values were used to compare observed and predicted toxicity in our study, rather than LC/EC50 values, predictions within a factor of 3 would also be considered reasonable (Peters et al. 2018).

### Applying the models to natural waters

Table 8 compares the PC95 and PC99 values for nickel data normalized using the 4 different models for each of the 20 natural waters covering a range of water chemistries. The PC90 and PC80 values for the trophic level‐specific MLR and the pooled MLR are given in the Supplemental Data, Table S5.

**Table 8:** Protective concentrations (PCs; 95 and 99% of species) for nickel for 20 natural waters after normalization with the 4 different models

	Trophic level‐specific MLR		Pooled MLR		European Union BLM		Soft water BLM	
Water	PC95	PC99	PC95	PC99	PC95	PC99	PC95	PC99
Australian waters								
Appletree Creek	22	3.9	220	78	28	20	11	6.8
Magela Creek	2.5	0.48	1.7	0.62	15	9.9	3.4	1.9
Ovens River	8.0	1.9	7.3	2.6	8.8	4.2	6.5	2.4
Ovens River adj.	1.3	0.17	3.9	1.4	0.94	0.25	0.84	0.2
Minamurra River	14	3.4	13	4.8	16	7.1	15	5.2
Tea Tree	11	2.7	10	3.6	22	9.8	18	6.4
Lake Eacham	3.6	0.85	4.0	1.4	4.1	1.6	2.8	0.87
Woronora River	5.9	1.4	4.4	1.5	8.4	3.3	6.8	2.3
Wellington	7.6	1.7	5.9	2.1	7.8	2.9	8.8	2.8
Sandy Creek	36	8.4	66	23	43	20	NC	NC
Milang	6.7	1.4	3.7	1.3	12	3.6	NC	NC
New Zealand waters								
Cascade Stream	2.2	0.32	5.3	1.9	1.8	0.54	NC	NC
Waipaoa River	4.8	0.77	3.0	1.1	4.4	1.3	NC	NC
Rangataiki River	5.0	1.2	3.9	1.4	5.4	2.3	NC	NC
Ohinemuri River	2.1	0.42	1.4	0.48	2.4	0.77	NC	NC
Hurunui River	2.4	0.49	0.95	0.33	2.2	0.69	NC	NC
Haast River	2.0	0.41	1.2	0.43	2.2	0.70	NC	NC
Mangapouri Stream	14	3.4	13	4.4	13	6.2	NC	NC
Porirua Stream	6.3	1.4	6.2	2.2	5.2	2.0	NC	NC
Mokotua Stream	31	2.0	2050	726	38	27	NC	NC
Carran Creek	28	5.5	36	13	35	20	NC	NC
Index condition	2.6	0.44	5.2	1.8	2.5	0.82	2.2	0.57

MLR = multiple linear regression; BLM = biotic ligand model; NC = not calculated.

As expected, water chemistry and choice of model strongly influenced the resulting protective concentration values. The largest range of PC95 and PC99 values (460‐fold) for water samples within model boundaries was predicted by the pooled MLR, followed by the European Union BLM (64‐fold difference in PC95 and 117‐fold difference in PC99 values) at the different water chemistry conditions. The PC95 values estimated using the trophic level‐specific MLR varied by 43‐fold, and the PC99 values varied by 65‐fold across the 20 different water chemistry combinations. The highest PC95 values, where water quality values were within the model limits, were for Sandy Creek for all models (43–66 µg Ni/L), that is, nickel in this water would be predicted to be the least bioavailable because it had a high DOC (21 mg/L, >90th percentile), relatively high hardness (117 mg CaCO_3_/L), and neutral pH (7.2). Nickel bioavailability was predicted by both the trophic level‐specific MLR and the European Union BLM to be the highest in 4 of the New Zealand waters, Cascade Stream, Hurunui River, and Haast River, which had low DOC (<2 mg/L), and Ohinemuri River, which had the highest pH of 8.3. These trends are in agreement with our previous understanding of the effects of pH, DOC, and hardness on nickel bioavailability (Peters et al. 2018).

Appletree Creek (pH 4.6) and Mokotua Stream (pH 4.4) were outside the model boundaries for pH. Not surprisingly, there was a large variation in the predicted protective concentration values for Appletree Creek between the models, with the pooled MLR giving a PC95 value 10 times higher than the trophic level‐specific MLR and BLMs. Similarly, for Mokotua Stream, the pooled MLR gave a PC95 value approximately 60 times higher than the trophic level‐specific MLR and the European Union BLM. For the soft Magela Creek water (hardness 2 mg CaCO_3_/L), the softwater BLM PC95 value was similar to both MLR PC95 values.

The Ovens River water was modeled at 2 pH values (6.9 and 8.2) and 2 DOC concentrations, keeping hardness constant, and, as expected, the PC95 values were lower at high pH and low DOC for all models, that is, all models were consistent in their predictions of increased nickel bioavailability.

Tea Tree had slightly higher pH and DOC, but lower hardness compared with Minamurra River. Both MLR models predicted lower PC95 values for Tea Tree, whereas both BLMs predicted higher PC95 values, suggesting that the BLMs were more influenced by DOC than by hardness or pH in this water. Milang had a very high pH (8.8) outside all the model upper pH boundaries, but with high hardness and high DOC. Again, the European Union BLM predicted a higher PC95 than the other models, possibly due to the greater amount of DOC data contributing to the DOC speciation in the Windermere Humic Aqueous Model (WHAM) underpinning this model. However, given the overlapping confidence limits in all the protective concentration values, this may not be a real difference.

All the models gave consistent predictions for the New Zealand natural waters. In higher pH waters, protective concentration values were lower than at lower pH, that is, nickel was more bioavailable and bioavailability decreased with increasing DOC and hardness. Although Waipaoa River had a high pH of 8.2 and relatively low DOC, its high hardness of 198 mg CaCO_3_/L was predicted to be protective of nickel toxicity. This was entirely consistent with our understanding of the effects of water chemistry on nickel bioavailability, and all models performed as expected with changing water chemistry.

Apart from the 2 softwaters (Magela Creek and Appletree Creek), there was good agreement between the European Union BLM and the softwater BLM for the 7 Australian waters tested in common. In agreement with Peters et al. (2018), either BLM could be applied to waters within the hardness range of 14 to 58 mg CaCO_3_/L.

No one model consistently predicted lower or higher protective concentration values than the other models. However, the pooled MLR did give the lowest PC95 values in 7 of the 10 Australian waters, whereas the trophic level‐specific MLR gave the lowest PC95 values in 3 of these waters. The trophic level‐specific MLR and the European Union BLM predicted similar PC95 values in 8 of the 10 Australian natural waters. For New Zealand waters, all models gave similar predictions of the PC95 values for all waters, except for the Mokotua River water, which had a very low pH outside all the model ranges.

## DISCUSSION

Ideally, the most appropriate bioavailability model for normalizing the ecotoxicity data would be based on a mechanistic understanding of nickel toxicity. The nickel BLMs have a mechanistic foundation based on a range of water chemistry parameters known to affect metal speciation and binding to biota. The MLRs are based on empirical relationships with a subset of these same water chemistry parameters, thereby retaining some mechanistic foundation. To this end, the applicability ranges of empirical MLR models should, in principle, be applied more stringently than those of the BLM because the models are not developed based on any mechanistic interpretation of the binding behavior of nickel either to ligands in solution or associated with the organisms. However, when BLMs and MLRs are compared, other factors including model accuracy and performance need to be considered, as well as the models' ease of use and how the model will be applied (Van Genderen et al. 2020). Final model selection will likely be a trade‐off among data and model input needs, model predictiveness and protectiveness, and desired applications.

The trophic level‐specific MLRs consistently scored the highest in the model assessment, using both the proportional and nonproportional approaches. They best predicted EC10 values, with 78% of predicted EC10 values within a factor of 2 of the observed EC10 values. This may be due to the large datasets for algae and invertebrates, and the fact that an additional local species, *H. viridissima*, was used in the MLR development, compared with its absence in the European Union BLM development. The relative errors for the trophic level‐specific MLRs were lower than for the pooled MLR and the BLMs, providing further support for selection of the trophic level‐specific MLR for bioavailability normalization (Peters et al. 2021).

However, the aquatic plant MLR and the fish MLR were based on only 2 species each and a limited number of tests with each covering a limited range of water chemistries. The MLR relationships for these were derived largely from tests in natural waters, so there may be confounding factors in such tests (e.g., unmeasured parameters). More chronic plant and fish data would improve our confidence in these 2 MLRs. The trophic level‐specific MLR is also relatively simple to use, because practitioners can calculate predicted EC10 values at their required water chemistry from the simple trophic‐level algorithms. In addition, look‐up tables, covering a range of typical water chemistry values, were developed and are presented in the Supplemental Data, Tables S6 to S9. One disadvantage of the trophic level‐specific MLR is that it does require each species in the nickel SSD to be corrected for its matching trophic‐level algorithm, compared with the use of the pooled MLR (e.g., as developed by Brix et al. 2017 for copper). However, the pooled MLR scored poorly in the model comparisons, and had poor adjusted and predictive *r* ^2^ values and higher relative errors, and only 66% of the data were within a factor of 2 of the observed EC10 values (Peters et al. 2021). For these reasons, we do not recommend use of the pooled MLR for nickel bioavailability normalization. The use of trophic level‐specific MLRs that normalize the entire SSD, is preferable, particularly when species from different trophic levels are the most sensitive species in the SSDs, as is the case for nickel.

The European Union BLM score was lower than the trophic level‐specific MLR, but it did reasonably predict EC10 values (68% within a factor of 2). Because the BLM is based on the WHAM 6 speciation model, which has been extensively validated for predicting nickel partitioning under thermodynamic conditions, the BLM may be considered a semimechanistic model compared with MLRs, which are based on empirical relationships only. However, use of the BLM is potentially more complex, although look‐up tables for guideline values can also be created at a range of different water chemistry conditions to improve its ease of use. A simplified tool, Bio‐met (2019) was developed to aid the process of checking compliance with the European Union nickel environmental quality standard. Bio‐met is essentially a look‐up table linked to PC95 values that are specific to the European Union databases and derivation process. Such a tool could be considered for Australia and New Zealand except it should be based on the quality ecotoxicity dataset we have used in the present study. This may assist in the BLM gaining acceptance by regulators in Australia and New Zealand.

The protective concentration values determined based on water chemistry data from typical Australia and New Zealand freshwater systems varied by >40‐fold, supporting the need for normalization of the nickel guideline values. At the index condition, protective concentration values derived with or without normalization gave similar values regardless of the model used for normalization. There was generally good agreement between the trophic level‐specific MLR and European Union BLM, in terms of both similar predicted protective concentration values and similar ranges of values (43–64‐fold) across the different water chemistries. Either of these models would be suitable to use for bioavailability normalization. The softwater BLM would be suitable for the soft waters for which it was designed (<10 mg CaCO_3_/L, but up to 50 CaCO_3_ mg/L; Peters et al. 2018), rather than being applied across the whole hardness range. In contrast, although the pooled MLR gave the most conservative protective concentration values in the natural waters, it also gave a much larger range of protective concentration values and more outliers and compared poorly with the other 3 models. The pooled MLR also gave less conservative protective concentration values at the index condition.

For application to slightly‐to‐moderately disturbed systems, a PC95 of 3 µg Ni/L would represent a relatively high nickel bioavailability condition and is appropriately conservative for a national guideline value. This value is the same as the PC95 values (2.7 and 2.5 µg Ni/L for unbounded and bounded datasets, respectively) from the larger dataset (44 species) with no bioavailability correction (Table 2). This is not surprising given that the majority of these nickel ecotoxicity data were generated from tests with low DOC (<10 mg/L), that is, conditions of relatively high bioavailability. The PC99, PC90, and PC80 values were also similar with or without bioavailability correction. The PC95 is above typical natural background concentrations of dissolved nickel found in freshwaters of 0.1 to 0.6 µg/L (Brix et al. 2017).

The PC95 of 3 µg Ni/L (at the index condition) is lower than the current nickel guideline value of 11 µg Ni/L (95% species protection, hardness adjusted to 30 mg CaCO_3_/L; Australian and New Zealand Governments 2018). The PC95 is similar to the bioavailability‐based nickel environmental quality standard in Europe, which ranges from 4 µg Ni/L for a reference condition up to a site‐specific value as high as 45 µg Ni/L. The PC99 of 0.4 µg Ni/L that we derived is also lower than the current nickel guideline value for high ecological value freshwaters (8 µg Ni/L). The PC99 value is within the background concentration of nickel for most freshwaters.

Using the trophic level‐specific MLRs, an additional 288 SSDs were run to derive freshwater nickel guideline values at a range of water chemistries. Predicted EC10 values for nickel for each species in the ecotoxicity database at pH 6.0, 6.5, 7.0, 7.5, 8.0, and 8.5, each at DOC concentrations of 0.5, 1, 3, 5, 10, and 20 mg/L, and at varying Ca (2, 4, 6, 7, 15, 30, 40, and 70 mg/L) and Mg (1.6, 3.1, 4, 5.3, 11, 22, 30, and 54 mg/L) were calculated and summarized in the Supplemental Data look‐up Tables S6 to S9. The PC95 guideline values varied with DOC by a factor of approximately 3 to 5 across the pH/hardness combinations, confirming that DOC has less of an effect on nickel toxicity than the large changes in guideline values as pH and hardness are varied (by up to 40‐fold).

Another consideration for model selection is whether the chosen model is under‐ or overconservative in its predictions. To ensure adequate ecosystem protection, it is preferable for the predicted toxicities to be the same as or higher (i.e., the EC10 values lower) than the observed EC10 values, not lower (i.e., the EC10 values higher) than the observed EC10 values. Despite the limited data, the algal and aquatic plant MLRs both predicted toxicity within a factor of 3 for the entire validation datasets, that is, neither one under‐ or overpredicted outside this factor of 3 (i.e., 9‐fold). The invertebrate and fish MLRs tended to slightly overpredict toxicity (i.e., gave lower EC10 values). The European Union and softwater BLMs slightly underpredicted toxicity (Peters et al. 2021). However, in the natural waters, where the whole SSD was used to derive protective concentration values, no model consistently gave the lowest protective concentration values. The European Union BLM has also been validated using natural waters in laboratory studies with an insect, snail, rotifer, and duckweed—species not included in the BLM development suite (Schlekat et al. 2010). These cross‐species extrapolations were tested in natural waters of varying water chemistries and gave reasonable predictions of EC50 and EC20 values. No such field validation has yet been undertaken for the new nickel MLRs.

A bias was noted by Peters et al. (2021) for both the algal and invertebrate MLRs, with overprediction of the EC10 values for the more sensitive EC10 values (i.e., less conservative) and underestimation of the EC10 values for the less sensitive EC10 values (more conservative). The tendency for overestimation of toxicity under insensitive conditions was greater than the tendency for underestimation of toxicity in sensitive conditions.

Although data from field and microcosm studies were not included in the ecotoxicity database used to derive or validate the MLRs, such supporting studies have recently been undertaken to test the protectiveness of BLM bioavailability‐normalized thresholds for nickel in other jurisdictions. Hommen et al. (2016) found that the no‐observed‐effect concentration for nickel in a freshwater aquatic microcosm containing phytoplankton, periphyton, zooplankton, and snails was 12 µg/L (based on the most sensitive species, the snail). This was approximately twice the hazardous concentration for 5% of species (HC5) derived from the chronic SSD at the same water chemistry. Nys et al. (2019) reported that the bioavailability‐based HC5 for nickel was also protective of freshwater microcosm community structure and function over 56 d under high DOC (14 mg/L) conditions. Some adverse effects on individual plankton species were observed, but these effects were transitory. Mebane et al. (2020b) exposed aquatic insect communities to nickel in artificial streams and found that taxa richness, total abundance, mayfly abundance, and periphyton (measured as chlorophyll a) decreased with increasing nickel concentrations over the range <0.1 to 1096 µg Ni/L. They combined laboratory and their artificial stream data into an SSD normalized for water chemistry and derived an HC5 of approximately 5 µg Ni/L. Takeshita et al. (2019) examined the relationships between benthic invertebrates and nickel concentrations at 45 field sites in Japan. They derived EC5 values for nickel corresponding to the concentration at which each of 3 indicators decreased by 5% from the 90th quantile value at the reference sites. Estimated EC5s were 0.2, 7.6, and 1.1 µg/L Ni (free ion concentration) for Ephemeroptera, Plecoptera, and Trichoptera richness, total wet biomass of all invertebrates, and total abundance of filter feeders. However, in a later study (Takeshita et al. 2020), the same authors cautioned against the use of such field data, due to the confounding factors of other contaminants (e.g., organics) also present.

These microcosm studies support the use of bioavailability‐based guideline values derived from chronic laboratory studies and SSDs, but suggest that the guideline values derived from the laboratory studies will be conservative, in keeping with the precautionary principle for environmental protection. The differences between guideline values derived from field and laboratory studies may also be due to the different taxonomic groups dominating each SSD, such as the prevalence of plant data in our tropical SSD and crustaceans in the temperate SSD. This provides further justification for combining the tropical and temperate datasets into one SSD to avoid these taxa biases.

## CONCLUSIONS

The present study is the first report of an MLR‐based bioavailability model being used to derive a guideline value for nickel for any jurisdiction worldwide. All the models, except the pooled MLR, provided similar predictions, and the small differences in the model predictions are unlikely to be ecologically significant. For freshwaters, we recommend that the new bioavailability‐based guideline values for nickel for the combined tropical and temperate dataset be submitted for adoption as default guideline values for Australia and New Zealand. For example, a guideline value of 3 µg/L for 95% species protection could be applied to slightly‐to‐moderately disturbed systems, and a 99% species protection guideline value for nickel of 0.4 µg/L could be applied with caution to pristine waters, noting that this is close to background concentrations for nickel in freshwaters Both of these guideline values apply at our high nickel bioavailability index condition only: pH 7.5, DOC 0.5 mg/L, and hardness 30 mg CaCO_3_/L (6 mg Ca/L and 4 mg Mg/L). This DOC value is lower than the 10th percentile of DOC values in Australia and New Zealand to ensure that the guideline value is appropriately conservative. Guideline values for natural waters covering the full range of water chemistries in Australia and New Zealand varied by 40‐fold, and these guideline values should also be conservative.

To derive site‐specific guideline values for nickel, we recommend that the trophic level‐specific MLRs for algae, aquatic plants, invertebrates, and fish be used (see Table 1). However, the aquatic plant MLR and fish MLR require further development with more species for validation. Using the trophic level‐specific MLRs was sufficiently conservative in that they generally slightly overpredicted toxicity, although there was a bias noted for underprediction at low EC10 values. Overprediction of toxicity, rather than underprediction, is preferable as part of a risk‐based approach, that is, it is not desirable to adopt an approach that might underprotect certain species because the models underpinning the guideline values overestimate the protective effect of the key water quality parameters (pH, hardness, and DOC) for some species.

## Supplemental Data

The Supplemental Data are available on the Wiley Online Library at https://doi.org/10.1002/etc.4885.

## Author Contributions Statement

J. Stauber conceived and led the project as well as scored the models and wrote the paper. A. Peters derived the MLRs and co‐wrote the paper. L. Golding, M. Adams, M. Binet, G. Batley, F. Gissi, K. McKnight, and J. Gadd undertook the research and data analyses. G. Merrington, E. Garman, E. Middleton, and C. Schlekat provided advice and funding throughout the project and reviewed the manuscript.

## Supporting information

This article includes online‐only Supplemental Data.

Supporting information.

Supporting information.

## Data Availability

Data, associated metadata, and calculation tools are available from the corresponding author (jenny.stauber@csiro.au).
