# Supplementary material for: Application of Bioavailability Models to Derive Chronic Guideline Values for Nickel in Freshwaters of Australia and New Zealand
Source: Environ Toxicol Chem. 2020 Nov 17;40(1):100–12. doi: 10.1002/etc.4885 (PMC7839744; doi:10.1002/etc.4885)
Supplement: Supplementary file 1 — Supporting information. [file ETC-40-100-s001.docx]

**Supplementary Information**

**Application of bioavailability models to derive guideline values for nickel in freshwaters of Australia and New Zealand**

Jenny Stauber^1^, Lisa Golding^1^, Adam Peters^2^, Graham Merrington^2^, Merrin Adams^1^, Monique Binet^1^, Graeme Batley^1^, Francesca Gissi^3^, Kitty McKnight^1^, Emily Garman^4^, Ellie Middleton^4^, Jenni Gadd^5^ and Chris Schlekat^4^

^1^ CSIRO Land and Water, Lucas Heights, NSW 2232 Australia

^2^ wca environment, Faringdon, Oxfordshire, United Kingdom

^3^ CSIRO Oceans and Atmosphere, Lucas Heights, NSW 2232 Australia

^4^ NiPERA Inc, Durham, NC, USA

^5^ National Institute of Water and Atmospheric Research (NIWA), Auckland, New Zealand

Table S1. Freshwater nickel ecotoxicity database for tropical and temperate species. (See attached excel spreadsheet)

Table S2. Water chemistry boundaries for each MLR for both the development datasets and the validation datasets (from Peters et al. in press)

| Model | Dataset | pH | | DOC (mg/L) | | Ca (mg/L) | |
| --- | --- | --- | --- | --- | --- | --- | --- |
|  |  | Lower | Upper | Lower | Upper | Lower | Upper |
| Algae | Development | 6.0 | 8.0 | 0.25 | 26 | 3 | 144 |
| Algae | Validation | 6.4 | 8.2 | 0.5 | 10 | 0.25 | 11 |
| Plants | Development | 6.9 | 8.3 | 0.7 | 7.1 | 3.4 | 72 |
| Plants | Validation | 6.4 | 8.2 | 0.5 | 10 | 0.25 | 11 |
| Invertebrates | Development | 5.9 | 8.2 | 0.25 | 17 | 0.1 | 88 |
| Invertebrates | Validation | 6.9 | 8.5 | 0.25 | 7.1 | 0.5 | 72 |
| Vertebrates | Development | 5.5 | 8.5 | 0.5 | 18 | 3.8 | 110 |
| Vertebrates | Validation | 6.4 | 8.2 | 0.25 | 10 | 0.25 | 11 |
| Pooled | Development | 5.5 | 8.5 | 0.25 | 26 | 0.1 | 110 |
| Pooled | Validation | 6.4 | 8.5 | 0.25 | 10 | 0.25 | 72 |

Table S3. Scores and ranking for all models for all ecotoxicity data using both the non-proportional and proportional approaches

Table S4. Scores and ranking for all models for ecotoxicity validation dataset only using both the non-proportional and proportional approaches

Table S5. Protective concentrations (80%, 90%, 95% and 99% of species) for nickel for 20 natural waters after normalization with the trophic-level specific and pooled MLRs

| Water | Trophic-level specific MLR | | | | Pooled MLR | | | |
| --- | --- | --- | --- | --- | --- | --- | --- | --- |
|  | PC80 | PC90 | PC95 | PC99 | PC80 | PC90 | PC95 | PC99 |
| Australian waters |  |  |  |  |  |  |  |  |
| Appletree Creek | 104 | 47 | 22 | 3.9 | 614 | 358 | 220 | 78 |
| Magela Creek | 10 | 5.0 | 2.5 | 0.48 | 4.7 | 2.8 | 1.7 | 0.62 |
| Ovens River | 28 | 15 | 8.0 | 1.9 | 20 | 12 | 7.3 | 2.6 |
| Ovens River adj. | 7.2 | 3.0 | 1.3 | 0.17 | 11 | 6.3 | 3.9 | 1.4 |
| Minamurra River | 51 | 27 | 14 | 3.4 | 38 | 22 | 13 | 4.8 |
| Tea Tree | 40 | 21 | 11 | 2.7 | 28 | 16 | 10 | 3.6 |
| Lake Eacham | 13 | 6.8 | 3.6 | 0.85 | 11 | 6.5 | 4.0 | 1.4 |
| Woronora River | 21 | 11 | 5.9 | 1.4 | 12 | 7.1 | 4.4 | 1.5 |
| Wellington | 29 | 15 | 7.6 | 1.7 | 16 | 9.6 | 5.9 | 2.1 |
| Sandy Creek | 127 | 67 | 36 | 8.4 | 184 | 107 | 66 | 23 |
| Milang | 27 | 13 | 6.7 | 1.4 | 10 | 6.0 | 3.7 | 1.3 |
| New Zealand waters |  |  |  |  |  |  |  |  |
| Cascade Stream | 11 | 4.9 | 2.2 | 0.32 | 15 | 8.7 | 5.3 | 1.9 |
| Waipaoa River | 24 | 11 | 4.8 | 0.77 | 8.4 | 4.9 | 3.0 | 1.1 |
| Rangataiki River | 18 | 9.3 | 5.0 | 1.2 | 11 | 6.3 | 3.9 | 1.4 |
| Ohinemuri River | 8.6 | 4.2 | 2.1 | 0.42 | 3.8 | 2.2 | 1.4 | 0.48 |
| Hurunui River | 9.3 | 4.7 | 2.4 | 0.49 | 2.6 | 1.5 | 0.95 | 0.33 |
| Haast River | 7.6 | 3.8 | 2.0 | 0.41 | 3.4 | 2.0 | 1.2 | 0.43 |
| Mangapouri Stream | 51 | 27 | 14 | 3.4 | 35 | 20 | 13 | 4.4 |
| Porirua Stream | 24 | 12 | 6.3 | 1.4 | 17 | 10 | 6.2 | 2.2 |
| Mokotua Stream | 346 | 104 | 31 | 2.0 | 5730 | 3330 | 2050 | 726 |
| Carran Creek | 118 | 58 | 28 | 5.5 | 100 | 58 | 36 | 13 |

Tables S6-S9. Nickel water quality guideline values for differing pH, hardness and DOC concentrations (µg/L)

Table S6. Guideline values (µg Ni/L) for protection of 99% of species

|  |  | | **pH 6.0** |  |  |  |  |  |  |  |
| --- | --- | --- | --- | --- | --- | --- | --- | --- | --- | --- |
|  | **Hardness** | | **12** | **23** | **31** | **39** | **83** | **166** | **223** | **397** |
|  | **Ca** | | **2** | **4** | **6** | **7** | **15** | **30** | **40** | **70** |
|  | **Mg** | | **1.6** | **3.1** | **4** | **5.3** | **11** | **22** | **30** | **54** |
| **DOC** | | **0.5** | 0.8 | 1.3 | 1.5 | 1.7 | 2.4 | 3.1 | 3.5 | 4.2 |
|  |  | **1** | 1.1 | 1.9 | 2.3 | 2.6 | 3.8 | 5.0 | 5.7 | 7.0 |
|  |  | **3** | 1.6 | 2.9 | 3.5 | 4.3 | 6.9 | 9.7 | 11 | 14 |
|  |  | **5** | 1.9 | 3.3 | 4.0 | 5.0 | 8.4 | 12 | 14 | 18 |
|  |  | **10** | 2.4 | 3.7 | 4.5 | 5.6 | 9.9 | 16 | 19 | 25 |
|  |  | **15** | 2.8 | 3.9 | 4.7 | 5.8 | 10 | 17 | 21 | 29 |
|  |  | **20** | 3.1 | 4.3 | 4.9 | 5.9 | 10 | 17 | 22 | 31 |

|  |  | | **pH 6.5** |  |  |  |  |  |  |  |
| --- | --- | --- | --- | --- | --- | --- | --- | --- | --- | --- |
|  | **Hardness** | | **12** | **23** | **31** | **39** | **83** | **166** | **223** | **397** |
|  | **Ca** | | **2** | **4** | **6** | **7** | **15** | **30** | **40** | **70** |
|  | **Mg** | | **1.6** | **3.1** | **4** | **5.3** | **11** | **22** | **30** | **54** |
| **DOC** | | **0.5** | 0.7 | 1.0 | 1.2 | 1.3 | 1.6 | 1.9 | 2.1 | 2.4 |
|  |  | **1** | 1.0 | 1.5 | 1.8 | 2.0 | 2.7 | 3.3 | 3.6 | 4.1 |
|  |  | **3** | 1.5 | 2.4 | 2.9 | 3.5 | 5.2 | 6.9 | 7.7 | 9.0 |
|  |  | **5** | 1.7 | 2.8 | 3.4 | 4.1 | 6.5 | 9.2 | 10 | 13 |
|  |  | **10** | 2.1 | 3.4 | 4.1 | 5.0 | 8.3 | 13 | 15 | 19 |
|  |  | **15** | 2.4 | 3.7 | 4.5 | 5.5 | 9.3 | 14 | 17 | 23 |
|  |  | **20** | 2.6 | 4.0 | 4.8 | 5.8 | 9.8 | 16 | 19 | 26 |

|  |  | | **pH 7.0** |  |  |  |  |  |  |  |
| --- | --- | --- | --- | --- | --- | --- | --- | --- | --- | --- |
|  | **Hardness** | | **12** | **23** | **31** | **39** | **83** | **166** | **223** | **397** |
|  | **Ca** | | **2** | **4** | **6** | **7** | **15** | **30** | **40** | **70** |
|  | **Mg** | | **1.6** | **3.1** | **4** | **5.3** | **11** | **22** | **30** | **54** |
| **DOC** | | **0.5** | 0.6 | 0.7 | 0.8 | 0.8 | 1.0 | 1.1 | 1.2 | 1.4 |
|  |  | **1** | 0.8 | 1.1 | 1.2 | 1.4 | 1.6 | 1.9 | 2.0 | 2.3 |
|  |  | **3** | 1.3 | 1.9 | 2.2 | 2.5 | 3.4 | 4.2 | 4.6 | 5.2 |
|  |  | **5** | 1.5 | 2.3 | 2.7 | 3.2 | 4.5 | 5.9 | 6.5 | 7.5 |
|  |  | **10** | 1.8 | 2.8 | 3.4 | 4.0 | 6.2 | 8.6 | 9.7 | 12 |
|  |  | **15** | 2.0 | 3.1 | 3.8 | 4.5 | 7.1 | 10 | 12 | 15 |
|  |  | **20** | 2.2 | 3.4 | 4.1 | 4.8 | 7.8 | 12 | 14 | 18 |

|  |  | | **pH 7.5** |  |  |  |  |  |  |  |
| --- | --- | --- | --- | --- | --- | --- | --- | --- | --- | --- |
|  | **Hardness** | | **12** | **23** | **31** | **39** | **83** | **166** | **223** | **397** |
|  | **Ca** | | **2** | **4** | **6** | **7** | **15** | **30** | **40** | **70** |
|  | **Mg** | | **1.6** | **3.1** | **4** | **5.3** | **11** | **22** | **30** | **54** |
| **DOC** | | **0.5** | 0.4 | 0.4 | 0.4 | 0.5 | 0.5 | 0.5 | 0.6 | 0.6 |
|  |  | **1** | 0.6 | 0.7 | 0.8 | 0.8 | 0.9 | 0.9 | 1.0 | 1.1 |
|  |  | **3** | 1.0 | 1.4 | 1.5 | 1.7 | 2.0 | 2.2 | 2.3 | 2.5 |
|  |  | **5** | 1.2 | 1.7 | 2.0 | 2.2 | 2.8 | 3.3 | 3.5 | 3.7 |
|  |  | **10** | 1.4 | 2.1 | 2.5 | 2.9 | 4.1 | 5.2 | 5.6 | 6.3 |
|  |  | **15** | 1.5 | 2.4 | 2.8 | 3.3 | 4.9 | 6.5 | 7.2 | 8.4 |
|  |  | **20** | 1.6 | 2.5 | 3.0 | 3.5 | 5.4 | 7.5 | 8.4 | 10.2 |

|  |  | | **pH 8.0** |  |  |  |  |  |  |  |
| --- | --- | --- | --- | --- | --- | --- | --- | --- | --- | --- |
|  | **Hardness** | | **12** | **23** | **31** | **39** | **83** | **166** | **223** | **397** |
|  | **Ca** | | **2** | **4** | **6** | **7** | **15** | **30** | **40** | **70** |
|  | **Mg** | | **1.6** | **3.1** | **4** | **5.3** | **11** | **22** | **30** | **54** |
| **DOC** | | **0.5** | 0.2 | 0.2 | 0.2 | 0.2 | 0.2 | 0.2 | 0.3 | 0.3 |
|  |  | **1** | 0.4 | 0.4 | 0.4 | 0.4 | 0.4 | 0.4 | 0.4 | 0.5 |
|  |  | **3** | 0.7 | 0.9 | 0.9 | 1.0 | 1.1 | 1.0 | 1.0 | 1.0 |
|  |  | **5** | 0.8 | 1.1 | 1.2 | 1.4 | 1.6 | 1.6 | 1.6 | 1.6 |
|  |  | **10** | 0.9 | 1.4 | 1.6 | 1.8 | 2.5 | 2.9 | 2.9 | 3.0 |
|  |  | **15** | 1.0 | 1.5 | 1.8 | 2.1 | 3.0 | 3.7 | 4.0 | 4.2 |
|  |  | **20** | 1.0 | 1.6 | 1.9 | 2.2 | 3.3 | 4.3 | 4.7 | 5.3 |

|  |  | | **pH 8.5** |  |  |  |  |  |  |  |
| --- | --- | --- | --- | --- | --- | --- | --- | --- | --- | --- |
|  | **Hardness** | | **12** | **23** | **31** | **39** | **83** | **166** | **223** | **397** |
|  | **Ca** | | **2** | **4** | **6** | **7** | **15** | **30** | **40** | **70** |
|  | **Mg** | | **1.6** | **3.1** | **4** | **5.3** | **11** | **22** | **30** | **54** |
| **DOC** | | **0.5** | 0.1 | 0.1 | 0.1 | 0.1 | 0.1 | 0.2 | 0.2 | 0.2 |
|  |  | **1** | 0.2 | 0.2 | 0.2 | 0.2 | 0.2 | 0.2 | 0.3 | 0.3 |
|  |  | **3** | 0.4 | 0.5 | 0.5 | 0.5 | 0.5 | 0.5 | 0.5 | 0.5 |
|  |  | **5** | 0.5 | 0.7 | 0.7 | 0.8 | 0.8 | 0.8 | 0.7 | 0.7 |
|  |  | **10** | 0.6 | 0.8 | 0.9 | 1.0 | 1.3 | 1.5 | 1.5 | 1.3 |
|  |  | **15** | 0.6 | 0.9 | 1.0 | 1.2 | 1.6 | 2.0 | 2.0 | 2.0 |
|  |  | **20** | 0.6 | 0.9 | 1.0 | 1.2 | 1.8 | 2.3 | 2.5 | 2.6 |

Table S7. Guideline values (µg Ni/L) for protection of 95% of species

|  |  | | **pH 6.0** |  |  |  |  |  |  |  |
| --- | --- | --- | --- | --- | --- | --- | --- | --- | --- | --- |
|  | **Hardness** | | **12** | **23** | **31** | **39** | **83** | **166** | **223** | **397** |
|  | **Ca** | | **2** | **4** | **6** | **7** | **15** | **30** | **40** | **70** |
|  | **Mg** | | **1.6** | **3.1** | **4** | **5.3** | **11** | **22** | **30** | **54** |
| **DOC** | | **0.5** | 3.6 | 5.3 | 6.2 | 7.0 | 9.8 | 13 | 15 | 19 |
|  |  | **1** | 5.1 | 7.9 | 9.3 | 11 | 15 | 21 | 24 | 30 |
|  |  | **3** | 8.4 | 13 | 16 | 19 | 29 | 40 | 46 | 59 |
|  |  | **5** | 10 | 16 | 19 | 23 | 36 | 53 | 61 | 79 |
|  |  | **10** | 14 | 21 | 25 | 29 | 47 | 72 | 84 | 112 |
|  |  | **15** | 16 | 23 | 28 | 33 | 54 | 83 | 99 | 134 |
|  |  | **20** | 18 | 26 | 30 | 36 | 58 | 90 | 109 | 149 |

|  |  | | **pH 6.5** |  |  |  |  |  |  |  |
| --- | --- | --- | --- | --- | --- | --- | --- | --- | --- | --- |
|  | **Hardness** | | **12** | **23** | **31** | **39** | **83** | **166** | **223** | **397** |
|  | **Ca** | | **2** | **4** | **6** | **7** | **15** | **30** | **40** | **70** |
|  | **Mg** | | **1.6** | **3.1** | **4** | **5.3** | **11** | **22** | **30** | **54** |
| **DOC** | | **0.5** | 3.1 | 4.3 | 5.0 | 5.5 | 7.5 | 9.8 | 11 | 14 |
|  |  | **1** | 4.4 | 6.3 | 7.4 | 8.3 | 12 | 15 | 17 | 21 |
|  |  | **3** | 6.9 | 11 | 12 | 14 | 21 | 29 | 33 | 42 |
|  |  | **5** | 8.4 | 13 | 15 | 18 | 27 | 38 | 44 | 56 |
|  |  | **10** | 11 | 17 | 20 | 23 | 36 | 53 | 62 | 80 |
|  |  | **15** | 13 | 19 | 23 | 27 | 42 | 63 | 74 | 99 |
|  |  | **20** | 14 | 21 | 25 | 30 | 47 | 70 | 83 | 113 |

|  |  | | **pH 7.0** |  |  |  |  |  |  |  |
| --- | --- | --- | --- | --- | --- | --- | --- | --- | --- | --- |
|  | **Hardness** | | **12** | **23** | **31** | **39** | **83** | **166** | **223** | **397** |
|  | **Ca** | | **2** | **4** | **6** | **7** | **15** | **30** | **40** | **70** |
|  | **Mg** | | **1.6** | **3.1** | **4** | **5.3** | **11** | **22** | **30** | **54** |
| **DOC** | | **0.5** | 2.5 | 3.3 | 3.7 | 4.1 | 5.3 | 6.9 | 7.7 | 9.6 |
|  |  | **1** | 3.5 | 4.8 | 5.4 | 6.0 | 8.1 | 10 | 12 | 14 |
|  |  | **3** | 5.5 | 8.0 | 9.3 | 11 | 15 | 20 | 22 | 27 |
|  |  | **5** | 6.7 | 9.8 | 12 | 13 | 19 | 26 | 29 | 36 |
|  |  | **10** | 8.4 | 13 | 15 | 17 | 26 | 36 | 42 | 53 |
|  |  | **15** | 9.7 | 15 | 17 | 20 | 30 | 44 | 51 | 66 |
|  |  | **20** | 11 | 16 | 19 | 22 | 34 | 49 | 57 | 75 |

|  |  | | **pH 7.5** |  |  |  |  |  |  |  |
| --- | --- | --- | --- | --- | --- | --- | --- | --- | --- | --- |
|  | **Hardness** | | **12** | **23** | **31** | **39** | **83** | **166** | **223** | **397** |
|  | **Ca** | | **2** | **4** | **6** | **7** | **15** | **30** | **40** | **70** |
|  | **Mg** | | **1.6** | **3.1** | **4** | **5.3** | **11** | **22** | **30** | **54** |
| **DOC** | | **0.5** | 1.8 | 2.3 | 2.6 | 2.8 | 3.5 | 4.4 | 4.9 | 5.9 |
|  |  | **1** | 2.6 | 3.4 | 3.8 | 4.1 | 5.2 | 6.5 | 7.2 | 8.7 |
|  |  | **3** | 4.2 | 5.7 | 6.5 | 7.3 | 9.6 | 12 | 13 | 16 |
|  |  | **5** | 5.0 | 7.1 | 8.1 | 9.1 | 13 | 16 | 18 | 22 |
|  |  | **10** | 6.2 | 9.0 | 11 | 12 | 17 | 23 | 26 | 32 |
|  |  | **15** | 6.9 | 10 | 12 | 14 | 20 | 28 | 32 | 40 |
|  |  | **20** | 7.5 | 11 | 13 | 15 | 22 | 32 | 36 | 46 |

|  |  | | **pH 8.0** |  |  |  |  |  |  |  |
| --- | --- | --- | --- | --- | --- | --- | --- | --- | --- | --- |
|  | **Hardness** | | **12** | **23** | **31** | **39** | **83** | **166** | **223** | **397** |
|  | **Ca** | | **2** | **4** | **6** | **7** | **15** | **30** | **40** | **70** |
|  | **Mg** | | **1.6** | **3.1** | **4** | **5.3** | **11** | **22** | **30** | **54** |
| **DOC** | | **0.5** | 1.3 | 1.6 | 1.7 | 1.8 | 2.1 | 2.7 | 3.0 | 3.8 |
|  |  | **1** | 1.8 | 2.3 | 2.5 | 2.7 | 3.2 | 3.8 | 4.1 | 5.1 |
|  |  | **3** | 2.9 | 3.9 | 4.3 | 4.7 | 6.0 | 7.1 | 7.6 | 8.8 |
|  |  | **5** | 3.4 | 4.7 | 5.4 | 6.0 | 7.8 | 9.6 | 10 | 12 |
|  |  | **10** | 4.1 | 5.9 | 6.9 | 7.7 | 11 | 14 | 15 | 18 |
|  |  | **15** | 4.4 | 6.5 | 7.7 | 8.7 | 13 | 17 | 19 | 22 |
|  |  | **20** | 4.6 | 6.9 | 8.2 | 9.4 | 14 | 19 | 21 | 26 |

|  |  | | **pH 8.5** |  |  |  |  |  |  |  |
| --- | --- | --- | --- | --- | --- | --- | --- | --- | --- | --- |
|  | **Hardness** | | **12** | **23** | **31** | **39** | **83** | **166** | **223** | **397** |
|  | **Ca** | | **2** | **4** | **6** | **7** | **15** | **30** | **40** | **70** |
|  | **Mg** | | **1.6** | **3.1** | **4** | **5.3** | **11** | **22** | **30** | **54** |
| **DOC** | | **0.5** | 0.9 | 1.0 | 1.1 | 1.1 | 1.5 | 2.0 | 2.2 | 2.7 |
|  |  | **1** | 1.2 | 1.5 | 1.6 | 1.7 | 1.9 | 2.5 | 2.8 | 3.6 |
|  |  | **3** | 1.9 | 2.5 | 2.8 | 3.0 | 3.6 | 4.0 | 4.3 | 5.3 |
|  |  | **5** | 2.2 | 3.0 | 3.4 | 3.7 | 4.7 | 5.5 | 5.8 | 6.6 |
|  |  | **10** | 2.5 | 3.6 | 4.1 | 4.6 | 6.3 | 8.0 | 8.6 | 9.7 |
|  |  | **15** | 2.6 | 3.8 | 4.5 | 5.1 | 7.2 | 9.5 | 11 | 12 |
|  |  | **20** | 2.6 | 3.9 | 4.7 | 5.3 | 7.8 | 11 | 12 | 14 |

Table S8. Guideline values (µg Ni/L) for protection of 90% of species

|  |  | | **pH 6.0** |  |  |  |  |  |  |  |
| --- | --- | --- | --- | --- | --- | --- | --- | --- | --- | --- |
|  | **Hardness** | | **12** | **23** | **31** | **39** | **83** | **166** | **223** | **397** |
|  | **Ca** | | **2** | **4** | **6** | **7** | **15** | **30** | **40** | **70** |
|  | **Mg** | | **1.6** | **3.1** | **4** | **5.3** | **11** | **22** | **30** | **54** |
| **DOC** | | **0.5** | 6.7 | 9.8 | 11 | 13 | 18 | 25 | 29 | 37 |
|  |  | **1** | 10 | 15 | 17 | 20 | 28 | 39 | 45 | 57 |
|  |  | **3** | 17 | 26 | 30 | 35 | 53 | 75 | 86 | 112 |
|  |  | **5** | 21 | 32 | 38 | 45 | 69 | 99 | 114 | 150 |
|  |  | **10** | 29 | 43 | 51 | 60 | 94 | 138 | 162 | 215 |
|  |  | **15** | 34 | 51 | 60 | 71 | 110 | 164 | 194 | 261 |
|  |  | **20** | 39 | 57 | 67 | 79 | 123 | 184 | 219 | 297 |

|  |  | | **pH 6.5** |  |  |  |  |  |  |  |
| --- | --- | --- | --- | --- | --- | --- | --- | --- | --- | --- |
|  | **Hardness** | | **12** | **23** | **31** | **39** | **83** | **166** | **223** | **397** |
|  | **Ca** | | **2** | **4** | **6** | **7** | **15** | **30** | **40** | **70** |
|  | **Mg** | | **1.6** | **3.1** | **4** | **5.3** | **11** | **22** | **30** | **54** |
| **DOC** | | **0.5** | 5.7 | 8.1 | 9.3 | 10 | 15 | 20 | 23 | 29 |
|  |  | **1** | 8.1 | 12 | 14 | 15 | 22 | 30 | 34 | 43 |
|  |  | **3** | 13 | 20 | 23 | 27 | 39 | 55 | 63 | 81 |
|  |  | **5** | 17 | 25 | 29 | 34 | 50 | 71 | 82 | 107 |
|  |  | **10** | 22 | 33 | 39 | 46 | 69 | 99 | 116 | 152 |
|  |  | **15** | 26 | 39 | 46 | 54 | 82 | 119 | 140 | 186 |
|  |  | **20** | 29 | 43 | 51 | 60 | 92 | 135 | 158 | 214 |

|  |  | | **pH 7.0** |  |  |  |  |  |  |  |
| --- | --- | --- | --- | --- | --- | --- | --- | --- | --- | --- |
|  | **Hardness** | | **12** | **23** | **31** | **39** | **83** | **166** | **223** | **397** |
|  | **Ca** | | **2** | **4** | **6** | **7** | **15** | **30** | **40** | **70** |
|  | **Mg** | | **1.6** | **3.1** | **4** | **5.3** | **11** | **22** | **30** | **54** |
| **DOC** | | **0.5** | 4.7 | 6.3 | 7.2 | 8.1 | 11 | 15 | 17 | 22 |
|  |  | **1** | 6.5 | 9.0 | 10 | 12 | 16 | 22 | 25 | 32 |
|  |  | **3** | 10 | 15 | 17 | 20 | 28 | 38 | 43 | 56 |
|  |  | **5** | 13 | 18 | 22 | 25 | 36 | 49 | 56 | 72 |
|  |  | **10** | 16 | 24 | 28 | 33 | 48 | 68 | 78 | 102 |
|  |  | **15** | 19 | 28 | 33 | 38 | 57 | 81 | 95 | 124 |
|  |  | **20** | 21 | 31 | 37 | 43 | 64 | 92 | 107 | 142 |

|  |  | | **pH 7.5** |  |  |  |  |  |  |  |
| --- | --- | --- | --- | --- | --- | --- | --- | --- | --- | --- |
|  | **Hardness** | | **12** | **23** | **31** | **39** | **83** | **166** | **223** | **397** |
|  | **Ca** | | **2** | **4** | **6** | **7** | **15** | **30** | **40** | **70** |
|  | **Mg** | | **1.6** | **3.1** | **4** | **5.3** | **11** | **22** | **30** | **54** |
| **DOC** | | **0.5** | 3.6 | 4.8 | 5.4 | 6.0 | 8.2 | 11 | 12 | 15 |
|  |  | **1** | 4.9 | 6.7 | 7.5 | 8.4 | 11 | 15 | 17 | 22 |
|  |  | **3** | 7.7 | 11 | 12 | 14 | 19 | 25 | 29 | 36 |
|  |  | **5** | 9.3 | 13 | 15 | 17 | 24 | 32 | 37 | 47 |
|  |  | **10** | 12 | 17 | 20 | 22 | 32 | 44 | 50 | 65 |
|  |  | **15** | 13 | 19 | 23 | 26 | 38 | 52 | 60 | 78 |
|  |  | **20** | 14 | 21 | 25 | 28 | 42 | 59 | 68 | 88 |

|  |  | | **pH 8.0** |  |  |  |  |  |  |  |
| --- | --- | --- | --- | --- | --- | --- | --- | --- | --- | --- |
|  | **Hardness** | | **12** | **23** | **31** | **39** | **83** | **166** | **223** | **397** |
|  | **Ca** | | **2** | **4** | **6** | **7** | **15** | **30** | **40** | **70** |
|  | **Mg** | | **1.6** | **3.1** | **4** | **5.3** | **11** | **22** | **30** | **54** |
| **DOC** | | **0.5** | 2.8 | 3.6 | 4.0 | 4.4 | 5.7 | 7.5 | 8.5 | 11 |
|  |  | **1** | 3.7 | 4.8 | 5.4 | 5.9 | 7.8 | 10 | 11 | 14 |
|  |  | **3** | 5.5 | 7.4 | 8.5 | 9.4 | 13 | 16 | 18 | 22 |
|  |  | **5** | 6.4 | 8.9 | 10 | 11 | 16 | 21 | 23 | 28 |
|  |  | **10** | 7.7 | 11 | 13 | 14 | 20 | 28 | 31 | 39 |
|  |  | **15** | 8.4 | 12 | 14 | 16 | 23 | 32 | 37 | 46 |
|  |  | **20** | 8.9 | 13 | 15 | 18 | 26 | 36 | 41 | 52 |

|  |  | | **pH 8.5** |  |  |  |  |  |  |  |
| --- | --- | --- | --- | --- | --- | --- | --- | --- | --- | --- |
|  | **Hardness** | | **12** | **23** | **31** | **39** | **83** | **166** | **223** | **397** |
|  | **Ca** | | **2** | **4** | **6** | **7** | **15** | **30** | **40** | **70** |
|  | **Mg** | | **1.6** | **3.1** | **4** | **5.3** | **11** | **22** | **30** | **54** |
| **DOC** | | **0.5** | 2.1 | 2.6 | 2.9 | 3.1 | 4.2 | 5.7 | 6.5 | 8.1 |
|  |  | **1** | 2.7 | 3.4 | 3.8 | 4.1 | 5.2 | 7.0 | 8.0 | 10 |
|  |  | **3** | 3.7 | 5.0 | 5.7 | 6.3 | 8.2 | 10 | 11 | 14 |
|  |  | **5** | 4.2 | 5.8 | 6.6 | 7.4 | 10 | 13 | 14 | 17 |
|  |  | **10** | 4.8 | 6.8 | 7.9 | 8.9 | 12 | 17 | 19 | 23 |
|  |  | **15** | 5.0 | 7.3 | 8.6 | 9.7 | 14 | 19 | 21 | 27 |
|  |  | **20** | 5.2 | 7.6 | 9.0 | 10 | 15 | 21 | 24 | 30 |

Table S9. Guideline values (µg Ni/L) for protection of 80% of species

|  |  | | **pH 6.0** |  |  |  |  |  |  |  |
| --- | --- | --- | --- | --- | --- | --- | --- | --- | --- | --- |
|  | **Hardness** | | **12** | **23** | **31** | **39** | **83** | **166** | **223** | **397** |
|  | **Ca** | | **2** | **4** | **6** | **7** | **15** | **30** | **40** | **70** |
|  | **Mg** | | **1.6** | **3.1** | **4** | **5.3** | **11** | **22** | **30** | **54** |
| **DOC** | | **0.5** | 13 | 18 | 21 | 24 | 35 | 49 | 56 | 73 |
|  |  | **1** | 19 | 28 | 32 | 37 | 53 | 74 | 86 | 113 |
|  |  | **3** | 34 | 50 | 59 | 68 | 100 | 141 | 164 | 216 |
|  |  | **5** | 44 | 65 | 76 | 88 | 131 | 187 | 218 | 289 |
|  |  | **10** | 61 | 91 | 107 | 124 | 186 | 269 | 315 | 421 |
|  |  | **15** | 74 | 110 | 130 | 151 | 228 | 330 | 387 | 520 |
|  |  | **20** | 84 | 126 | 148 | 173 | 262 | 381 | 447 | 601 |

|  |  | | **pH 6.5** |  |  |  |  |  |  |  |
| --- | --- | --- | --- | --- | --- | --- | --- | --- | --- | --- |
|  | **Hardness** | | **12** | **23** | **31** | **39** | **83** | **166** | **223** | **397** |
|  | **Ca** | | **2** | **4** | **6** | **7** | **15** | **30** | **40** | **70** |
|  | **Mg** | | **1.6** | **3.1** | **4** | **5.3** | **11** | **22** | **30** | **54** |
| **DOC** | | **0.5** | 11 | 15 | 18 | 20 | 29 | 41 | 47 | 62 |
|  |  | **1** | 15 | 22 | 25 | 29 | 42 | 59 | 68 | 90 |
|  |  | **3** | 26 | 38 | 44 | 51 | 74 | 104 | 121 | 160 |
|  |  | **5** | 33 | 48 | 56 | 65 | 95 | 135 | 156 | 208 |
|  |  | **10** | 45 | 66 | 78 | 90 | 132 | 189 | 220 | 292 |
|  |  | **15** | 54 | 79 | 93 | 107 | 160 | 229 | 267 | 357 |
|  |  | **20** | 61 | 90 | 105 | 122 | 182 | 262 | 306 | 411 |

|  |  | | **pH 7.0** |  |  |  |  |  |  |  |
| --- | --- | --- | --- | --- | --- | --- | --- | --- | --- | --- |
|  | **Hardness** | | **12** | **23** | **31** | **39** | **83** | **166** | **223** | **397** |
|  | **Ca** | | **2** | **4** | **6** | **7** | **15** | **30** | **40** | **70** |
|  | **Mg** | | **1.6** | **3.1** | **4** | **5.3** | **11** | **22** | **30** | **54** |
| **DOC** | | **0.5** | 8.9 | 12 | 14 | 16 | 24 | 33 | 39 | 51 |
|  |  | **1** | 12 | 17 | 20 | 22 | 32 | 46 | 53 | 70 |
|  |  | **3** | 20 | 28 | 33 | 37 | 53 | 75 | 87 | 116 |
|  |  | **5** | 25 | 35 | 41 | 46 | 67 | 94 | 110 | 146 |
|  |  | **10** | 32 | 47 | 55 | 62 | 91 | 129 | 150 | 199 |
|  |  | **15** | 38 | 55 | 64 | 74 | 108 | 154 | 179 | 238 |
|  |  | **20** | 42 | 62 | 72 | 83 | 122 | 174 | 203 | 270 |

|  |  | | **pH 7.5** |  |  |  |  |  |  |  |
| --- | --- | --- | --- | --- | --- | --- | --- | --- | --- | --- |
|  | **Hardness** | | **12** | **23** | **31** | **39** | **83** | **166** | **223** | **397** |
|  | **Ca** | | **2** | **4** | **6** | **7** | **15** | **30** | **40** | **70** |
|  | **Mg** | | **1.6** | **3.1** | **4** | **5.3** | **11** | **22** | **30** | **54** |
| **DOC** | | **0.5** | 7.3 | 10 | 12 | 13 | 19 | 27 | 31 | 41 |
|  |  | **1** | 9.5 | 13 | 15 | 17 | 25 | 35 | 40 | 54 |
|  |  | **3** | 15 | 20 | 23 | 27 | 38 | 54 | 62 | 82 |
|  |  | **5** | 18 | 25 | 29 | 32 | 46 | 65 | 76 | 100 |
|  |  | **10** | 22 | 32 | 37 | 42 | 61 | 85 | 99 | 131 |
|  |  | **15** | 26 | 37 | 43 | 49 | 71 | 100 | 116 | 154 |
|  |  | **20** | 28 | 40 | 47 | 54 | 79 | 111 | 129 | 172 |

|  |  | | **pH 8.0** |  |  |  |  |  |  |  |
| --- | --- | --- | --- | --- | --- | --- | --- | --- | --- | --- |
|  | **Hardness** | | **12** | **23** | **31** | **39** | **83** | **166** | **223** | **397** |
|  | **Ca** | | **2** | **4** | **6** | **7** | **15** | **30** | **40** | **70** |
|  | **Mg** | | **1.6** | **3.1** | **4** | **5.3** | **11** | **22** | **30** | **54** |
| **DOC** | | **0.5** | 6.0 | 8.2 | 9.5 | 11 | 15 | 21 | 24 | 32 |
|  |  | **1** | 7.4 | 10 | 12 | 13 | 19 | 26 | 30 | 40 |
|  |  | **3** | 10 | 15 | 17 | 19 | 27 | 38 | 44 | 57 |
|  |  | **5** | 12 | 17 | 20 | 22 | 32 | 44 | 51 | 68 |
|  |  | **10** | 15 | 21 | 24 | 27 | 40 | 55 | 64 | 85 |
|  |  | **15** | 16 | 23 | 27 | 31 | 45 | 63 | 73 | 96 |
|  |  | **20** | 18 | 25 | 30 | 34 | 49 | 69 | 80 | 106 |

|  |  | | **pH 8.5** |  |  |  |  |  |  |  |
| --- | --- | --- | --- | --- | --- | --- | --- | --- | --- | --- |
|  | **Hardness** | | **12** | **23** | **31** | **39** | **83** | **166** | **223** | **397** |
|  | **Ca** | | **2** | **4** | **6** | **7** | **15** | **30** | **40** | **70** |
|  | **Mg** | | **1.6** | **3.1** | **4** | **5.3** | **11** | **22** | **30** | **54** |
| **DOC** | | **0.5** | 4.9 | 6.7 | 7.6 | 8.5 | 12 | 17 | 19 | 24 |
|  |  | **1** | 5.8 | 7.9 | 9.1 | 10 | 14 | 20 | 23 | 30 |
|  |  | **3** | 7.4 | 10 | 12 | 13 | 19 | 26 | 30 | 39 |
|  |  | **5** | 8.2 | 12 | 13 | 15 | 21 | 30 | 34 | 45 |
|  |  | **10** | 9.4 | 13 | 16 | 18 | 25 | 35 | 41 | 53 |
|  |  | **15** | 10 | 14 | 17 | 19 | 28 | 39 | 45 | 59 |
|  |  | **20** | 11 | 15 | 18 | 20 | 29 | 41 | 48 | 63 |


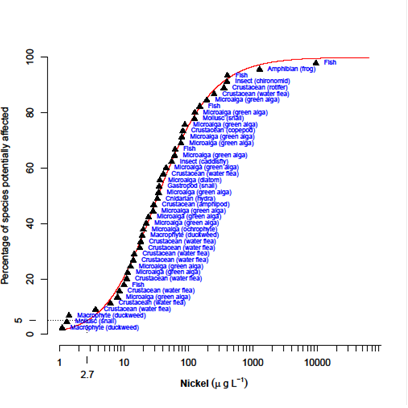


C


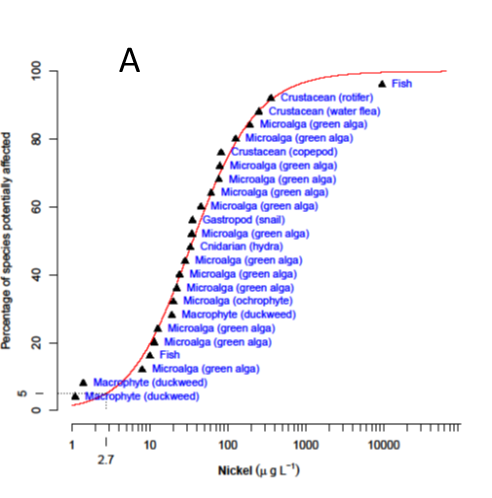

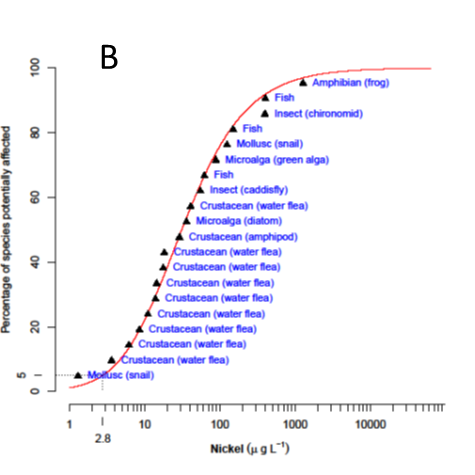


Figure S1. SSDs for unbounded non-normalised data showing tropical (A), temperate (B) and combined tropical and temperate (C) species


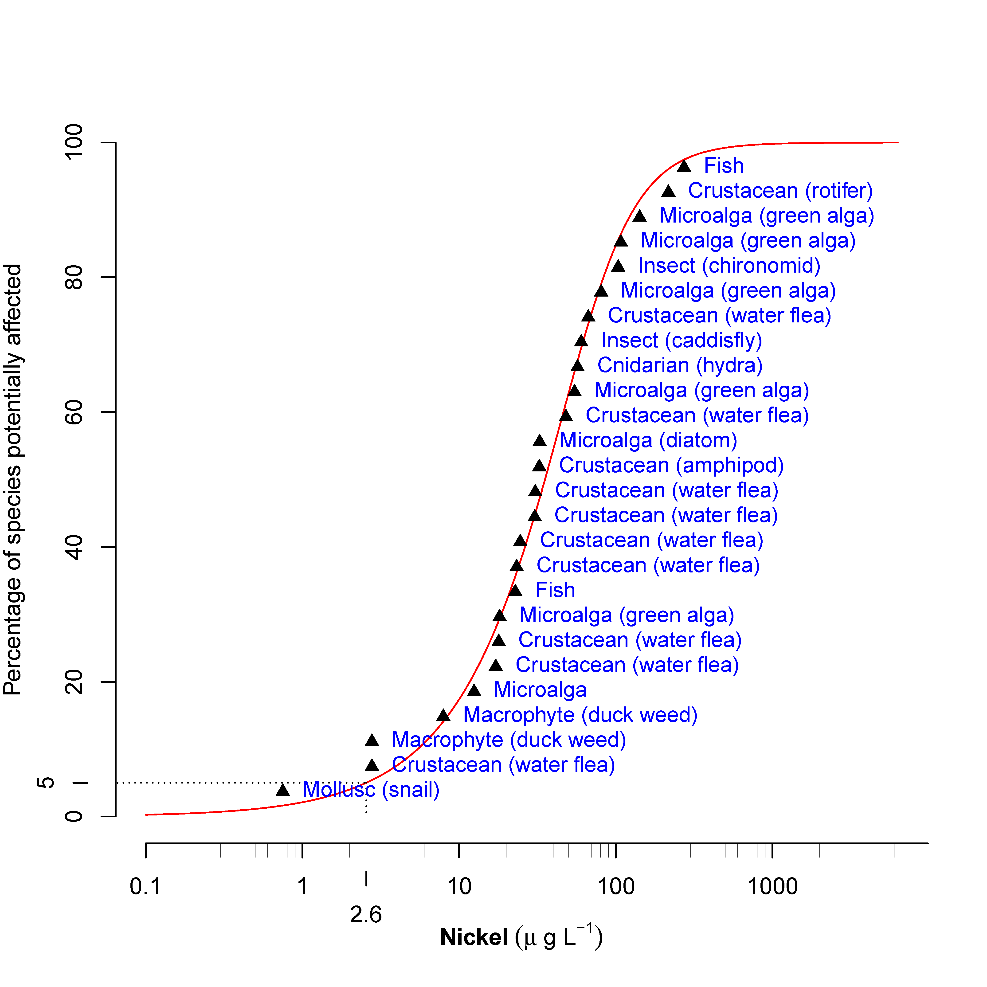


Figure S2. Species sensitivity distribution of freshwater nickel chronic toxicity for combined temperate and tropical data, normalised to the index condition using the trophic level-specific MLRs
